# LOMIX, a Mixture of Flaxseed Linusorbs, Exerts Anti-Inflammatory Effects through Src and Syk in the NF-κB Pathway

**DOI:** 10.3390/biom10060859

**Published:** 2020-06-04

**Authors:** Zubair Ahmed Ratan, Deok Jeong, Nak Yoon Sung, Youn Young Shim, Martin J. T. Reaney, Young-Su Yi, Jae Youl Cho

**Affiliations:** 1 Department of Integrative Biotechnology, Biomedical Institute for Convergence at SKKU (BICS), Sungkyunkwan University, Suwon 16419, Korea; zubairahmed@bme.kuet.ac.bd (Z.A.R.); jd279601@gmail.com (D.J.); nakyoon.sung@monash.edu (N.Y.S.); younyoung.shim@usask.ca (Y.Y.S.); 2 Department of Biomedical Engineering, Khulna University of Engineering and Technology, Khulna 9203, Bangladesh; 3 Department of Plant Sciences, University of Saskatchewan, Saskatoon, SK S7N 5A8, Canada; 4 Guangdong Saskatchewan Oilseed Joint Laboratory, Department of Food Science and Engineering, Jian University, Guangzhou 510632, China; 5 Department of Life Sciences, Kyonggi University, Suwon 16227, Korea

**Keywords:** flaxseed oil, linusorb, LOMIX, anti-inflammatory, Src, Syk, NF-κB

## Abstract

Although flax (*Linum usitatissimum* L.) has long been used as Ayurvedic medicine, its anti-inflammatory role is still unclear. Therefore, we aimed to investigate the anti-inflammatory role of a linusorb mixture (LOMIX) recovered from flaxseed oil. Effects of LOMIX on inflammation and its mechanism of action were examined using several in vitro assays (i.e., NO production, real-time PCR analysis, luciferase-reporter assay, Western blot analysis, and kinase assay) and in vivo analysis with animal inflammation models as well as acute toxicity test. *Results:* LOMIX inhibited NO production, cell shape change, and inflammatory gene expression in stimulated RAW264.7 cells through direct targeting of Src and Syk in the NF-κB pathway. In vivo study further showed that LOMIX alleviated symptoms of gastritis, colitis, and hepatitis in murine model systems. In accordance with in vitro results, the in vivo anti-inflammatory effects were mediated by inhibition of Src and Syk. LOMIX was neither cytotoxic nor did it cause acute toxicity in mice. In addition, it was found that LOB3, LOB2, and LOA2 are active components included in LOMIX, as assessed by NO assay. These in vitro and in vivo results suggest that LOMIX exerts an anti-inflammatory effect by inhibiting the inflammatory responses of macrophages and ameliorating symptoms of inflammatory diseases without acute toxicity and is a promising anti-inflammatory medication for inflammatory diseases.

## 1. Introduction

Inflammation mediated by the innate immune system protects the body against various invading pathogens and cellular danger through signals [1,2,3,4]. An inflammatory response is induced by recognition of pathogen-associated molecular patterns (PAMPs) and danger-associated molecular patterns (DAMPs) via pattern recognition receptors (PRRs) [1,2,5], with the type of inflammatory response dependent on PRR type. Inflammatory responses mediated by extracellular Toll-like receptors (TLRs) activate only the priming process, a preparatory step for inflammatory responses. In the priming process, inflammatory signaling pathways such as nuclear factor-kappa B (NF-κB), activated protein-1 (AP-1), and interferon (IFN) regulatory factor (IRF) are activated, leading to production and secretion of a variety of inflammatory mediators and cytokines such as nitric oxide (NO), prostaglandin E_2_ (PGE_2_), tumor necrosis factor (TNF)-α, interleukin (IL)-1β and IL-6, IFN-β, and IP-10 [6,7,8,9,10,11]. Inflammatory responses mediated by interaction of PAMPs and DAMPs with intracellular PRRs, such as nucleotide-binding oligomerization domain (NOD)-like receptors (NLRs), RIG-I-like receptors (RLRs), absent in melanoma 2 (AIM2)-like receptors (ALRs), and caspase-11 activate the triggering process. During this process, inflammasomes are formed and activated, leading to gasdermin D (GSDMD)-mediated pyroptosis (an inflammatory form of cell death) and caspase-1-mediated secretion of IL-1β and IL-18 [3,4,12,13].

Flax (*Linum usitatissimum* L.) has been grown for food, fiber, and oil in temperate climates for thousands of years and also used in Ayurvedic medicines [14]. Flaxseed has long been utilized as the source of flaxseed oil, one of the oldest commercial oils and a good source of various bioactive compounds such as omega-3 fatty acids, alpha linolenic acid, and the lignan secoisolariciresinol diglucoside [14]. Flaxseed is consumed both as a dietary supplement to improve human health and as medication to alleviate symptoms of various human diseases including cardiovascular disease, diabetes, neuronal disease, menopausal symptoms, skin disease, gastrointestinal disease, and even cancer [14]. Although the pharmacological activity of flaxseed and flaxseed fractions have been investigated, the pharmacological role of flaxseed compounds in the inflammatory response remains poorly understood. Therefore, in this study, an anti-inflammatory role of a linusorb mixture (LOMIX, also known as cyclolinopeptide mixture) was investigated using an in vitro macrophage model and several in vivo mouse models of inflammatory diseases.

## 2. Materials and Methods

### 2.1. Materials

**Figure 1:**
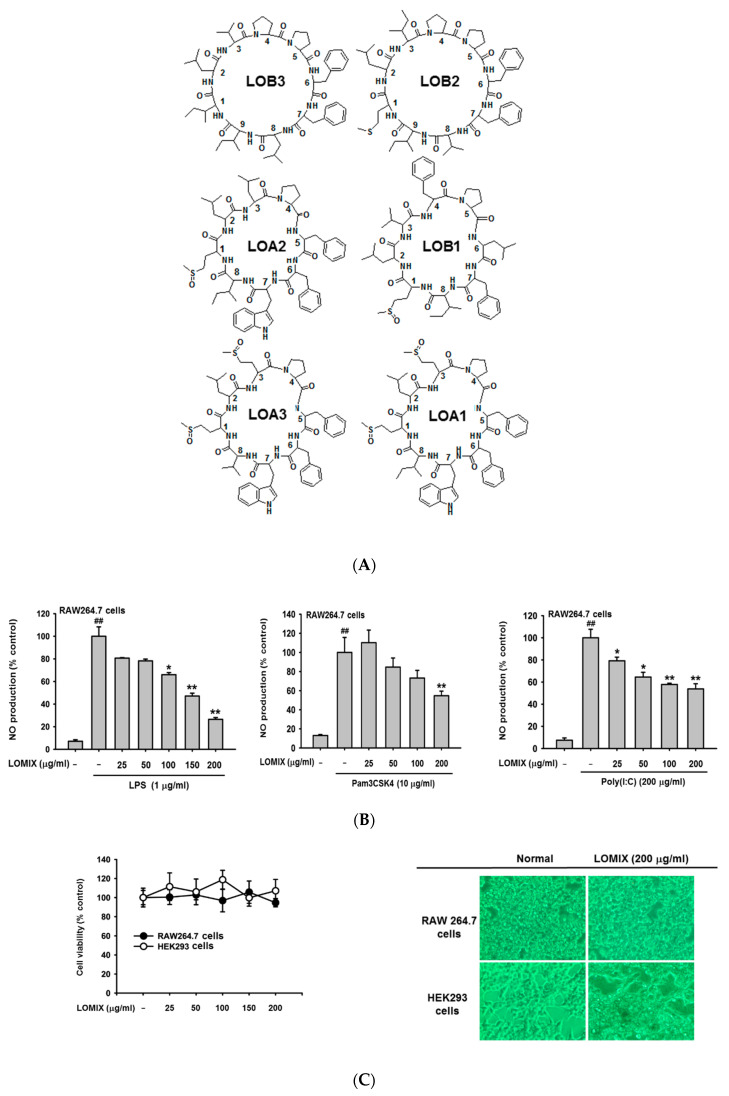

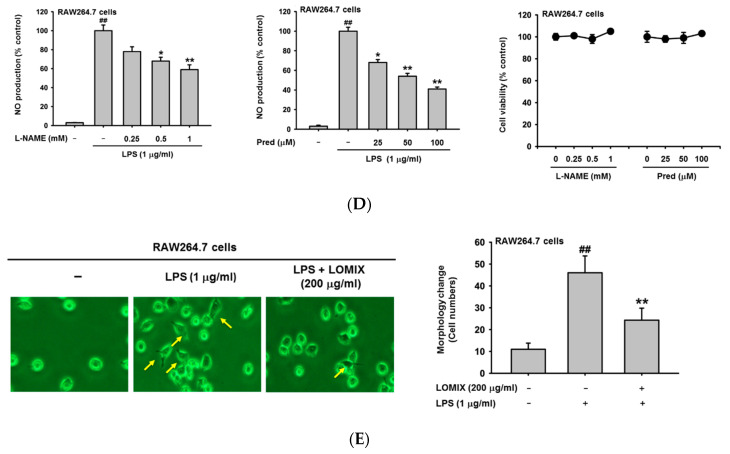
Linusorb mixture (LOMIX) has anti-inflammatory activity in macrophages. (**A**) Chemical structures of LOMIX components. (**B**) RAW264.7 cells pretreated with the indicated doses of LOMIX for 30 min were treated with either LPS (1 µg/mL), Pam3CSK4 (10 µg/mL), or Poly(I:C) (200 µg/mL) for 24 h. The NO level in cell culture media was determined by Griess assay. (**C**) RAW264.7 and HEK293 cells were treated with the indicated doses of LOMIX for 24 h, and the cell viability was determined by MTT assay (left panel). Photos were taken with a digital camera (right panel). (**D**) RAW264.7 cells pretreated with the indicated doses of either (**D left**) L-NAME or (**D middle**) Pred for 30 min were treated with LPS (1 µg/mL) for 24 h, and NO level in the cell culture media was determined by Griess assay. (**D right**) RAW264.7 cells were treated with the indicated doses of either L-NAME or Pred for 24 h, and the cell viability was determined by MTT assay. RAW264.7 cells pretreated with LOMIX (200 µg/mL) for 30 min were treated with LPS (1 µg/mL) for 24 h. (**E left**) Cell shape was photographed, and (**E right**) the number of cells with an altered shape was counted using a hemocytometer and plotted. ^##^ *p* < 0.01 compared to the vehicle-treated control. * *p* < 0.05, ** *p* < 0.01 compared to the stimulator-treated controls. Original magnification of (**E**) was 250×.

LOMIX (Figure 1A and Table 1) was provided as a generous contribution from Prairie Tide Diversified Inc. (Saskatchewan, SK, Canada). ICR mice (male, 6-weeks-old, 20–25 g) were purchased from Dae Han Bio Link Co., Ltd. (Osong, Korea), and a pelleted diet was purchased from Samyang Co. (Daejeon, Korea). RAW264.7 and HEK293 cells were purchased from the American Type Culture Collection (Rockville, MD, USA). Dulbecco’s Modified Eagle’s Medium (DMEM), Roswell Park Memorial Institute (RPMI) 1640 medium, fetal bovine serum (FBS), phosphate-buffered saline (PBS), streptomycin, penicillin, L-glutamine, and MuLV reverse transcriptase (RT) and protein ladder were purchased from Thermo Fisher Scientific (Waltham, MA, USA). Lipopolysaccharide (LPS), Pam3CSK4, and Poly (I:C) were purchased from InvivoGen (Pak Shek Kok, Hong Kong). N(ω)-nitro-L-arginine methyl ester (L-NAME), prednisolone (Pred), 3-(4,5-dimethylthiazol-2-yl)-2,5-diphenyltetrazolium bromide (MTT), dimethyl sulfoxide (DMSO), polyethylenimine (PEI), ATP, ranitidine, hematoxylin–eosin staining solution, dextran sulfate sodium salt (DSS), and D-galactosamine (D-GalN) were purchased from Sigma Chemical Co. (St. Louis, MO, USA). TRI reagent^®^ was purchased from Molecular Research Center Inc. (Cincinnati, OH, USA). Primers for quantitative real-time polymerase chain reaction (PCR) and RT-PCR were synthesized at Bioneer Inc. (Daejeon, Korea). Antibodies for Western blot analysis were purchased from Cell Signaling Technology (Beverly, MA, USA) and Santa Cruz Biotechnology (Santa Cruz, CA, USA). Enhanced chemiluminescence (ECL) reagents were purchased from AbFrontier Co., Ltd. (Seoul, Korea). Plasmids were purchased from Addgene (Watertown, MA, USA). Aspartate transaminase (AST) and alanine transaminase (ALT) ELISA kits were purchased from Abcam (Cambridge, UK).

### 2.2. Animal Husbandry

Mice (ICR) were housed in cages with a 12-h light-dark cycle. They were fed a pelleted diet and tap water *ad libitum*. Animal care and studies using the mice were conducted according to guidelines and protocols approved by the Sungkyunkwan University Animal Care and Use Committee.

### 2.3. Cell Culture

RAW264.7 and HEK293 cells were cultured at 37 °C in a humidified incubator with 5% CO_2_ in RPMI 1640 and DMEM, respectively, supplemented with 10% heat-inactivated FBS, penicillin (100 U/mL), streptomycin (100 mg/mL), and L-glutamine (2 mM). Cells were split 2–3 times per week, and the passage of cells used in this study was between 10 and 15.

### 2.4. Cell Viability Assay

RAW264.7 and HEK293 cells were treated with either LOMIX (0–200 g/mL), L-NAME (0–1 mM), Pred (0–100 µM), or individual linusorb for 24 h, and cell viability was determined by MTT assay as described previously [15]. Photos of these cells treated with LOMIX were taken by a digital camera.

### 2.5. NO Production Assay

RAW264.7 cells pretreated with either LOMIX (0–200 µg/mL), L-NAME (0–1 mM), Pred (0–100 µM), or individual linusorb for 30 min were treated with TLR ligands (10 µg/mL Pam3CSK4, 200 µg/mL poly I:C, or 1 µg/mL LPS) for 24 h. The amount of NO secreted into the cell culture media was determined by Griess assay as described previously [16].

### 2.6. Quantitative Real-Time PCR and Semiquantitative RT-PCR

RAW264.7 cells were pretreated with LOMIX (0–200 µg/mL) for 30 min followed by treatment with LPS (1 µg/mL) for 6 h. Total RNA was extracted from cells using TRI reagent^®^, and cDNA was immediately synthesized from total RNA (1 µg) using MuLV RT according to the manufacturer’s instructions. The cDNA was used for quantitative real-time PCR and semiquantitative RT-PCR to detect mRNA levels of iNOS, COX-2, and TNF-α [17]. Primer sequences used in this study are listed in Table 1.

### 2.7. Luciferase Reporter Gene Assay

HEK293 cells were cotransfected with NF-κB-luciferase reporter gene plasmids, β-galactosidase plasmids, and either empty pcDNA, MyD88, or TRIF plasmids for 24 h using PEI and then, treated with LOMIX (0–200 µg/mL) for another 24 h [18]. Cells were then lysed by three cycles of freezing (−70 °C) and thawing, and luciferin was added to the cell lysates. Luciferase reporter gene activity was determined by measuring luminescence and was normalized to that of β-galactosidase.

### 2.8. Western Blot Analysis

RAW264.7 cells pretreated with the indicated doses of LOMIX for 30 min were treated with LPS (1 µg/mL) for the indicated times. HEK293 cells transfected with the indicated plasmids for 24 h were treated with the indicated doses of LOMIX for another 24 h. Whole cell and nuclear lysates for Western blot analysis were prepared as described previously [19,20]. Stomach lysates of mice with gastritis (see below), liver lysates of mice with hepatitis (see below), and organ lysates of mice administered LOMIX (50 or 200 mg/kg) were prepared using a tissue homogenizer in ice-cold lysis buffer (20 mM Tris HCl, pH 7.4, 2 mM EDTA, 2 mM ethyleneglycotetraacetic acid, 50 mM β-glycerophosphate, 1 mM sodium orthovanadate, 1 mM dithiothreitol, 1% Triton X-100, 10% glycerol, 10 mg/mL aprotinin, 10 mg/mL pepstatin, 1 mM benzimide, and 2 mM PMSF) with rotation at 4 °C for 30 min. Whole cell, nuclear, and mouse organ lysates were subjected to Western blot analyses using antibodies specific for each protein as described previously [21].

### 2.9. In Vitro Kinase Assay

Src and Syk were purified, immunoprecipitated, and then subjected to an in vitro kinase assay using a kinase profiler service (Millipore, Billerica, MA, USA). Briefly, purified kinases were incubated with Mg and radiolabeled [γ^−32^ P] ATP at room temperature for 40 min, followed by addition of 3% phosphoric acid to stop the reaction. Each reaction solution (10 µL) was spotted onto a filter mat, and the mat was washed three times with phosphoric acid (75 mM) for 5 min each time and then rinsed once with methanol. The spotted reaction product was dried, and the incorporation of radiolabeled [γ^−32^ P] ATP was determined with a scintillation counter as a kinase activity.

### 2.10. HCl/EtOH-Induced Gastritis in Mice

Gastritis was induced in mice using HCl/EtOH as described previously [22]. Briefly, mice received either LOMIX (50 or 200 mg/kg) or ranitidine (40 mg/kg) orally twice a day for 3 days. Thirty minutes after the last administration, gastritis was induced by oral administration of a HCl/EtOH solution (150 mM HCl in 60% EtOH), and the stomachs of the mice were excised 1 h after gastritis induction. Stomachs were washed five times with PBS and cut along the greater curvature to observe the inside. Gastric lesions were photographed, and areas of ulcerative lesions were measured with ImageJ software (NIH, MD, USA) by a person blinded to the experiments. Stomach lysates were also subjected to Western blot analysis.

### 2.11. DSS-Induced Colitis in Mice

Colitis was induced in mice using DSS as described previously [23]. Briefly, mice received LOMIX (50 or 200 mg/kg) orally twice a day for 3 days. Thirty minutes after the last administration, colitis was induced by oral administration of 3% DSS (v/v), and colons were excised 7 days after colitis induction. Colons were washed five times with PBS, and colon length was measured. Colon tissues were also subjected to histological analysis by hematoxylin and eosin (H&E) staining.

### 2.12. LPS/D-GalN-Induced Hepatitis in Mice

Hepatitis was induced in mice using LPS and D-GalN as described previously [24]. Briefly, mice received LOMIX (50 or 200 mg/kg) orally twice a day for 3 days. Thirty minutes after the last administration, hepatitis was induced in mice by oral administration of LPS (10 µg/kg) and D-GalN (1 mg/kg), and livers were excised 6 h after hepatitis induction. Livers were washed five times with PBS and subjected to histological analysis by H&E staining. Blood was collected from the mice to evaluate serum levels of AST and ALT by a Roche Modular spectrophotometric autoanalyzer. Liver lysates were also subjected to Western blot analysis.

### 2.13. In Vivo Acute Toxicity Test

Mice received a single dose of LOMIX (5 g/kg) orally, and 24 h later, their organs (brain, thymus, heart, lung, liver, spleen, stomach, kidney, bladder, and colon) were excised. The appearance and weight of excised organs were compared with those of the control mice. Lysates of the organs were also subjected to Western blot analysis, and serum levels of AST and ALT were measured by a Roche Modular spectrophotometric autoanalyzer.

### 2.14. Statistical Analysis

All data in this study are expressed as the mean ± standard error (SE) of three independent sets of experimental data performed with at least three samples. Animal experiments were carried out with 5 to 8 mice per testing group. The statistical significance of differences between groups was determined by the Mann–Whitney *u* test, and a *p*-value < 0.05 was considered to indicate a statistically significant difference. All statistical analyses were performed using SPSS software (SPSS Inc., Chicago, IL, USA).

## 3. Results and Discussion

### 3.1. LOMIX Has Anti-Inflammatory Activity in Macrophages

Anti-inflammatory activity of LOMIX was first evaluated by determining inhibition of NO production in RAW264.7 cells, as NO production is a central sign of the inflammatory response [25]. For this, we tested LOMIX up to 200 µg/mL, since LOMIX is a mixture of six different cyclolinopeptides and mixtures or extracts are normally tested at concentrations ranging from 150 to 200 µg/mL [26,27]. Treatment with LOMIX significantly inhibited NO production in RAW264.7 cells stimulated with either Pam3CSK4 (10 µg/mL), Poly (I:C) (200 µg/mL), or LPS (1 µg/mL), molecular ligands of TLR2, TLR3, and TLR4, respectively, in a dose-dependent manner (Figure 1B). Although an agent might have good pharmacological activity, it cannot be developed into a therapeutic drug if it is cytotoxic or has adverse effects. Therefore, prior to investigating the anti-inflammatory activity of LOMIX, we first evaluated its cytotoxicity in RAW264.7 cells and HEK293 cells. LOMIX did not show cytotoxicity in the two cell types at any dose tested, according to MTT assay and morphological shapes of cells (Figure 1C left and right panels). Similar to LOMIX, L-NAME (0–1 mM) and Pred (0–100 µM), known anti-inflammatory agents, also inhibited NO production in LPS-stimulated RAW264.7 cells in a dose-dependent manner without significant cytotoxicity (Figure 1D), suggesting that LOMIX has comparable anti-inflammatory effect to the currently used anti-inflammatory agents. Macrophages polarized toward pro-inflammatory (M1) phenotypes undergo extensive changes in cell shape and adopt a dendritic-like morphology with large filopodia [28,29]. Therefore, the anti-inflammatory activity of LOMIX was further evaluated by determining the ability of LOMIX to inhibit LPS-induced morphological changes of macrophages. LPS induced marked morphological changes in RAW264.7 cells, whereas LOMIX significantly inhibited the LPS-induced morphological changes in RAW264.7 cells (Figure 1E). Taken together, these findings indicate that LOMIX exert an anti-inflammatory activity by inhibiting NO production and M1-polarized morphological changes in macrophages without significant cytotoxicity at the doses tested in this study.

### 3.2. LOMIX Suppresses mRNA Expression of Inflammatory Genes by Inhibiting NF-κB

**Figure 2:**
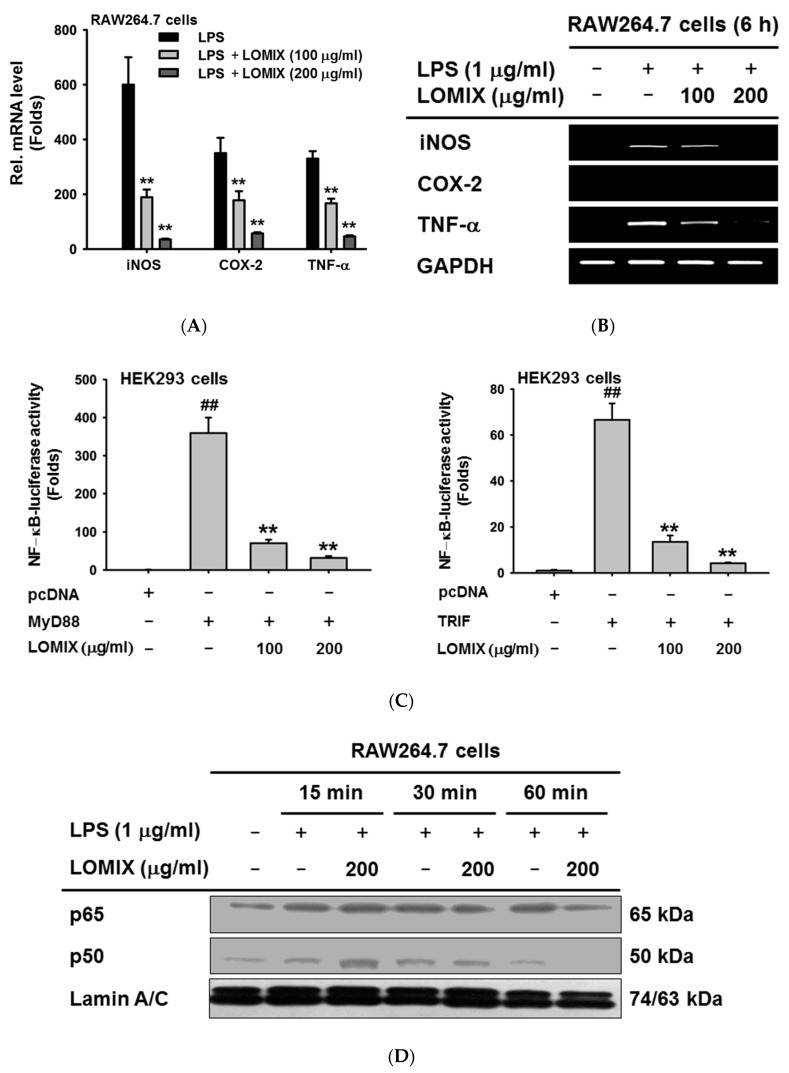
LOMIX suppresses mRNA expression of inflammatory genes by inhibiting NF-κB. RAW264.7 cells pretreated with the indicated doses of LOMIX for 30 min were treated with LPS (1 µg/mL) for 6 h, and mRNA expression levels of iNOS, COX-2, and TNF-α were determined by (**A**) quantitative real-time PCR and (**B**) semiquantitative RT-PCR. (**C**) HEK293 cells pretreated with the indicated doses of LOMIX for 24 h were transfected with the indicated plasmids for another 24 h. NF-κB luciferase reporter gene activity was determined by luminometer and normalized to that of β-galactosidase. (**D**) RAW264.7 cells pretreated with LOMIX (200 µg/mL) for 30 min were treated with LPS (1 µg/mL) for the indicated time. p65 and p50 in nuclear lysates were detected by Western blot analysis, and lamin A/C was used as an internal control. ^##^ *p* < 0.01 compared to the vehicle-treated control. ** *p* < 0.01 compared to the stimulator-treated controls.

During the inflammatory response, the expression of various inflammatory enzymes and pro-inflammatory cytokines, such as inducible NO synthase (iNOS), cyclooxygenase (COX)-2, TNF-α, IL-1β, IL-6, IFN-β, and IP-10, increases markedly [6,7,8,9]. Therefore, we evaluated whether LOMIX suppressed expression of these inflammatory enzymes and pro-inflammatory cytokines during response to inflammatory treatments. Quantitative real-time PCR showed that LOMIX significantly suppressed the LPS-induced increase in mRNA expression of iNOS, COX-2, and TNF-α in RAW264.7 cells in a dose-dependent manner (Figure 2A). Semiquantitative RT-PCR confirmed this result that LOMIX markedly suppressed the LPS-induced increase in mRNA expression of iNOS, COX-2, and TNF-α in RAW264.7 cells (Figure 2B). We next investigated how LOMIX suppresses expression of inflammatory genes. Several signal transduction pathways are activated during the inflammatory response, among which the NF-κB signaling pathway is one of the most critical, leading to production of inflammatory mediators and upregulation of inflammatory genes in macrophages [6,7,8,9]. Therefore, we investigated if LOMIX inhibited NF-κB activation during the inflammatory response. Luciferase reporter gene assay clearly revealed that in a dose-dependent manner, LOMIX significantly inhibited transcriptional activity of NF-κB induced by MyD88 and TRIF, critical adaptor molecules involved in the TLR-mediated inflammatory responses [7] (Figure 2C). Transcription factors translocate into the nucleus for transcriptional activity; therefore, we examined if LOMIX inhibited nuclear translocation of NF-κB transcription factors in macrophages. Western blot analysis showed that LOMIX inhibited nuclear translocation of the NF-κB transcription factors p65 and p50 at 60 min in LPS-stimulated RAW264.7 cells (Figure 2D). These results suggest that LOMIX inhibits expression of inflammatory genes by suppressing the activity of NF-κB transcription factors in macrophages.

### 3.3. LOMIX Suppresses the NF-κB Signaling Pathway

**Figure 3:**
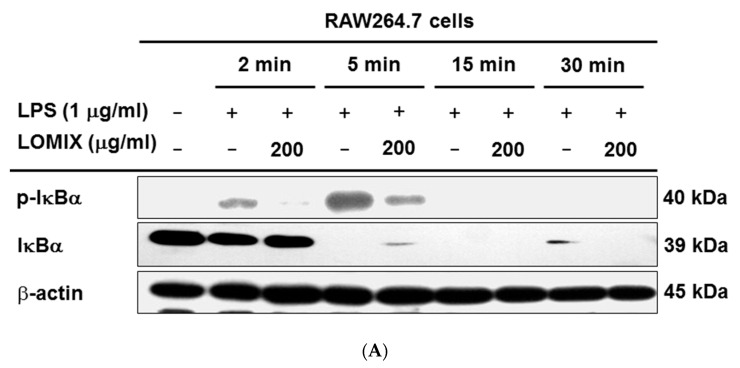

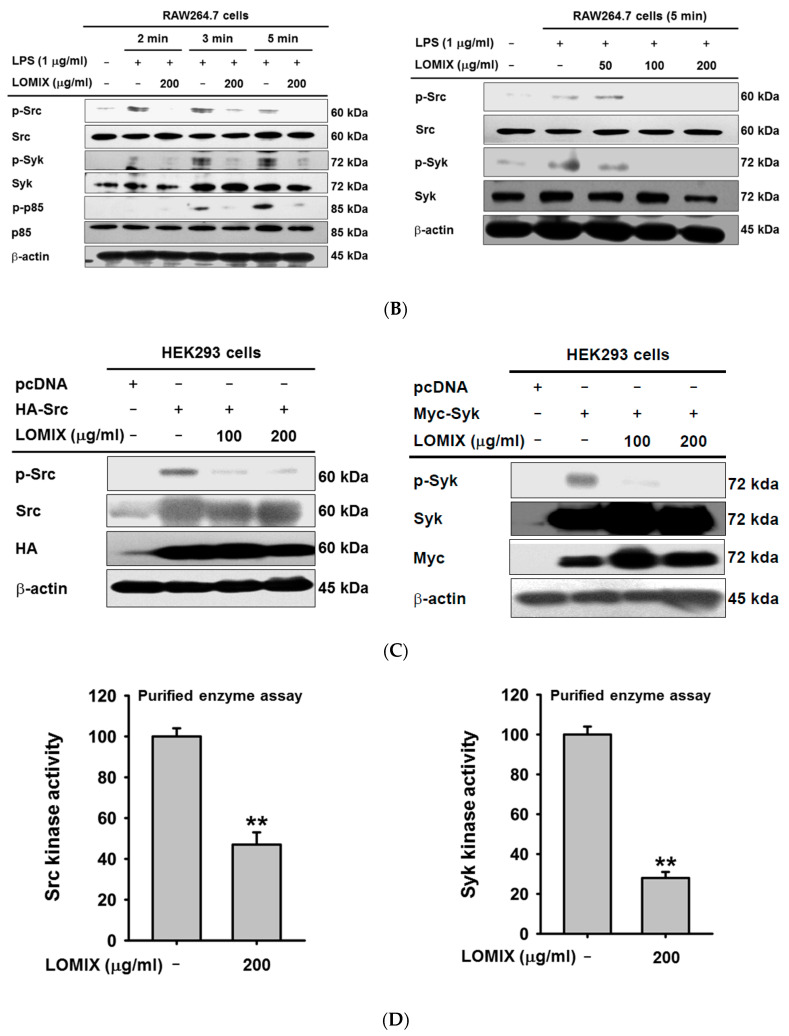
LOMIX suppresses NF-κB signaling. RAW264.7 cells pretreated with LOMIX (200 µg/mL) for 30 min were treated with LPS (1 µg/mL) for the indicated time. (**A**) p-IκBα and IκBα in whole cell lysates were detected by Western blot analysis. (**B left**) RAW264.7 cells pretreated with LOMIX (200 µg/mL) for 30 min were treated with LPS (1 µg/mL) for the indicated time, and p-Src, Src, p-Syk, Syk, p-p85, and p85 in whole cell lysates were detected by Western blot analysis. (**B right**) RAW264.7 cells pretreated with the indicated doses of LOMIX for 30 min were treated with LPS (1 µg/mL) for 5 min, and p-Src, Src, p-Syk, and Syk in whole cell lysates were detected by Western blot analysis. (**C**) HEK293 cells pretreated with the indicated doses of LOMIX for 24 h were transfected with the indicated plasmids for another 24 h, and p-Src, Src, p-Syk, Syk, and hemagglutinin (HA) in whole cell lysates were detected by Western blot analysis. β-actin and lamin A/C were used as internal controls for whole cell and nuclear lysates, respectively. (**D**) Inhibitory activity of LOMIX on Src and Syk kinase activities was determined by an in vitro kinase assay with purified enzymes. ** *p* < 0.01 compared to the vehicle-treated control.

A variety of intracellular signaling molecules, including Src, Syk, p85, AKT, inhibitory kappa B kinases (IKKs), and inhibitor of kappa B α (IκBα), are activated in macrophages during the inflammatory responses [7]. Because LOMIX suppressed the activity of NF-κB transcription factors, we evaluated if LOMIX inhibited intracellular signaling molecules in the NF-κB signaling pathway. In LPS-stimulated RAW264.7 cells, LOMIX markedly suppressed phosphorylation of IκBα, an upstream kinase that activates NF-κB transcription factors (Figure 3A). LOMIX also suppressed the phosphorylation of kinases upstream of IκBα such as Src, Syk, and p85 in LPS-stimulated RAW264.7 cells (Figure 3B left panel), in addition to the phosphorylation of Src and Syk in a dose-dependent manner (Figure 3B right panel). These results were further confirmed by transfecting HEK293 cells with constructs expressing these kinases. HEK293 cells were transfected with either Src-expressing or Syk-expressing constructs, and the effects of LOMIX on phosphorylation of both kinases was evaluated. LOMIX markedly inhibited the phosphorylation of both Src (Figure 3C left panel) and Syk (Figure 3C right panel), indicating that LOMIX suppresses activation of Src and Syk as well as their downstream kinase, p85. Although LOMIX showed marked suppression of Src and Syk in macrophages during the LPS-stimulated inflammatory response, it was not clear if Src and Syk were direct targets of LOMIX. To examine this, we conducted an in vitro kinase assay using purified Src and Syk; the results clearly showed that LOMIX decreased the kinase activity of Src and Syk (Figure 3D), strongly indicating that both Src and Syk are direct targets of LOMIX. These results suggest that LOMIX suppresses the activation of various inflammatory kinases in the NF-κB signaling pathway, and that Src and Syk are direct molecular targets of LOMIX, resulting in suppression of the NF-κB signaling pathway in macrophages during the inflammatory response.

### 3.4. LOMIX Ameliorates Gastritis Symptoms in a Mouse Model of Gastritis, a DSS-Induced Mouse Model of Colitis, and an LPS/D-GalN-Induced Mouse Model of Hepatitis

**Figure 4:**
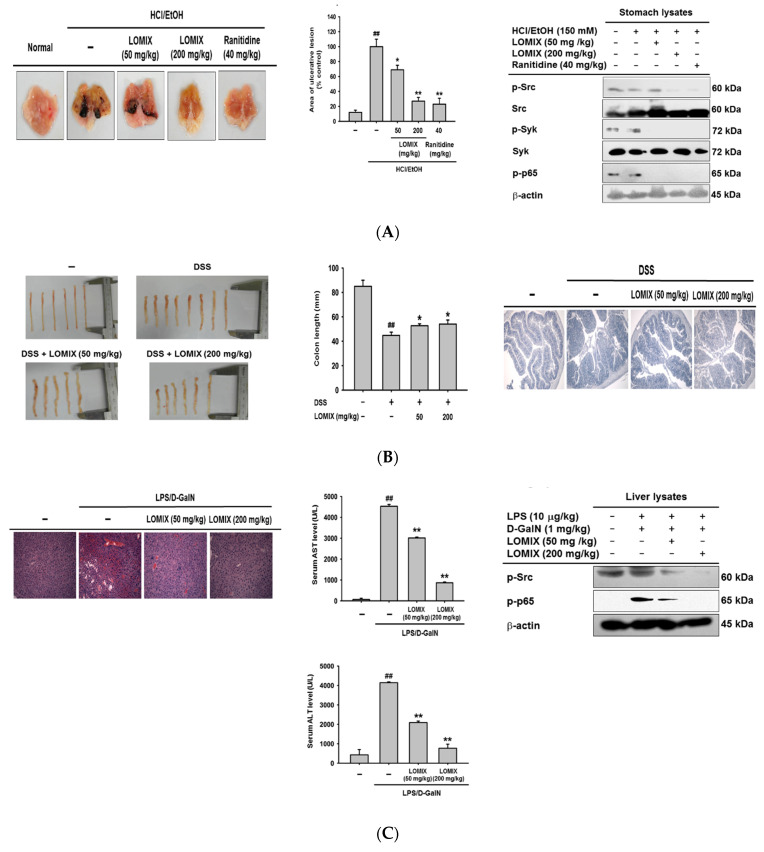
LOMIX ameliorates the symptoms of inflammatory diseases in mice. (**A**) ICR mice received the indicated doses of LOMIX or ranitidine (40 mg/kg) orally twice a day for 3 days. Thirty minutes after the final administration, experimental gastritis was induced by administering HCl/EtOH to the mice for 1 h. (**A left**) Ulcerative gastritis lesions were photographed, and (**A middle**) the areas of gastritis lesions were measured using ImageJ software and plotted. (**A right**) p-Src, Src, p-Syk, Syk, and p-p65 in whole stomach lysates were detected by Western blot analysis. (**B**) ICR mice received the indicated doses of LOMIX orally twice a day for 3 days. Thirty minutes after the final administration, experimental colitis was induced by administering 3% DSS (v/v) to the mice for 7 days. (**B left**) Colons were photographed, and (**B middle**) colon length was measured using calipers and plotted. (**B right**) Colon tissue was stained with H&E for histological analysis. (**C**) ICR mice received the indicated doses of LOMIX orally twice a day for 3 days. Thirty minutes after the final administration, experimental hepatitis was induced by administering LPS (10 µg/kg) and D-GalN (1 mg/kg) to the mice for 6 h. (**C left**) Liver tissues were subjected to H&E staining for histological analysis. (**C middle**) Serum levels of AST and ALT were determined by ELISA. (**C right**) p-Src and p-p65 in whole liver lysates were detected by Western blot analysis. β-actin was used as an internal control. ^##^ *p* < 0.01 compared to the vehicle-treated control. * *p* < 0.05, ** *p* < 0.01 compared to the stimulator-treated controls.

To determine if the anti-inflammatory effect observed in vitro also occurred in vivo, we used various well-established animal models of inflammatory diseases such as gastritis, colitis, and hepatitis [23,24,30,31]. We first investigated the anti-inflammatory effect of LOMIX in an HCl/EtOH-induced gastritis mouse model because gastric ulcer disease has had a tremendous effect on global morbidity and mortality until fairly recently [32]. Treatment with HCl/EtOH induced severe gastritis in control mice, but LOMIX reduced the HCl/EtOH-induced gastric ulcers in a dose-dependent manner (Figure 4A left). The area of gastric ulcerative lesions was also measured and was significantly decreased by LOMIX (Figure 4A middle). Interestingly, the suppressive effect of LOMIX in the pathogenesis of gastritis was comparable to that of ranitidine, an FDA-approved antiulcer medication (Figure 4A left & middle). Given that LOMIX was not cytotoxic (Figure 1C), LOMIX could be a promising antiulcer medication with comparable pharmacological effects and better safety than the current top-selling medication, ranitidine. To investigate the molecular mechanism by which LOMIX exerted its antigastritic effects in vivo, we evaluated the anti-inflammatory effect of LOMIX on Src and Syk in the NF-κB pathway [7,33] in stomach tissue. LOMIX markedly inhibited phosphorylation of both Src and Syk (Figure 4A right). These results are consistent with the in vitro anti-inflammatory effect of LOMIX in RAW264.7 cells (Figure 3B,C). Moreover, LOMIX suppressed phosphorylation of the NF-κB transcription factor p65 (Figure 4A right), which along with its translocation to the nucleus, is important for activation of this transcription factor. These results demonstrate that LOMIX had a strong in vivo anti-inflammatory effect and ameliorated gastritis symptoms by inhibiting the activation of Src and Syk, which are critical inflammatory molecules in the NF-κB signaling pathway, as well as activation of p65, an NF-κB transcription factor.

Inflammatory bowel diseases, such as Crohn’s disease and ulcerative colitis, are chronic diseases that cause inflammation of the gastrointestinal tract. Inflammatory bowel diseases are global diseases in the 21st century and are strongly associated with increased morbidity and mortality and represent a financial burden to the healthcare system [34,35]. Therefore, we examined the anti-inflammatory effect of LOMIX in the pathogenesis of ulcerative colitis using a DSS-induced colitis mouse model, which is a popular animal model for study of ulcerative colitis [36]. DSS treatment markedly shortened colon length, whereas LOMIX suppressed DSS-induced colon shortening in mice (Figure 4B left). Colon length was plotted to confirm the preventive effect of LOMIX on DSS-induced colon shortening in mice (Figure 4B middle). Given that one of the cardinal features of ulcerative colitis is inflammation-induced colon shortening, our finding that LOMIX inhibited colon shortening by suppressing colon inflammation suggested that LOMIX might have a protective effect against ulcerative colitis [23,37]. Another feature of ulcerative colitis is inflammation-induced damage of colon tissue [37]. Therefore, we evaluated the effect of LOMIX on DSS-damaged colon tissue. H&E staining showed that DSS-induced colon tissue damage was inhibited by LOMIX in a dose-dependent manner (Figure 4B right). These results suggest that LOMIX ameliorates colitis symptoms by inhibiting shortening of the colon and colon tissue damage.

Hepatitis is an inflammatory disease of the liver caused by various factors including toxins, medications, alcohol, autoimmunity, and viruses [38]. More than 1 million hepatitis patients die every year [39]. Therefore, we investigated the in vivo anti-inflammatory effect of LOMIX using an LPS/D-GalN-induced hepatitis mouse model [24]. H&E staining showed that LOMIX prevented LPS/D-GalN-induced liver tissue damage (Figure 4C left), indicating that LOMIX alleviates hepatitis symptoms by inhibiting liver tissue damage. Elevation of liver enzyme levels in the sera is a signal of liver inflammation and damage [40], and serum levels of liver enzymes such as AST and ALT have been shown to be elevated in the LPS/D-GalN-induced hepatitis mouse model [24]. We found that LOMIX significantly reduced serum levels of AST and ALT in LPS/D-GalN-induced hepatitis mice in a dose-dependent manner (Figure 4C middle). Similarly, LOMIX suppressed phosphorylation of Src and p65 in liver tissue of LPS/D-GalN-induced hepatitis mice (Figure 4C right). These results suggest that LOMIX ameliorated the symptoms of hepatitis in the mouse model by preventing inflammation-induced liver damage and decreasing serum levels of the liver enzymes AST and ALT, which are elevated when liver tissue is damaged. These liver-protective effects of LOMIX were mediated by suppression of activation of Src and p65, a critical inflammatory molecule and transcription factor in the NF-κB signaling pathway, respectively.

### 3.5. In Vivo Acute Toxicity of LOMIX

**Figure 5:**
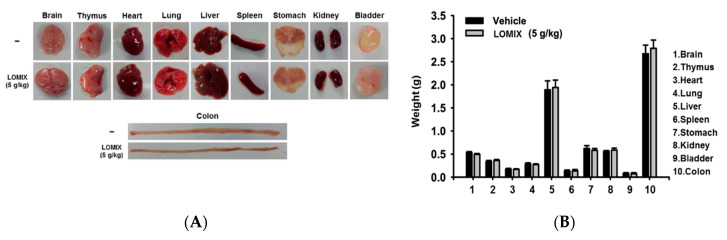

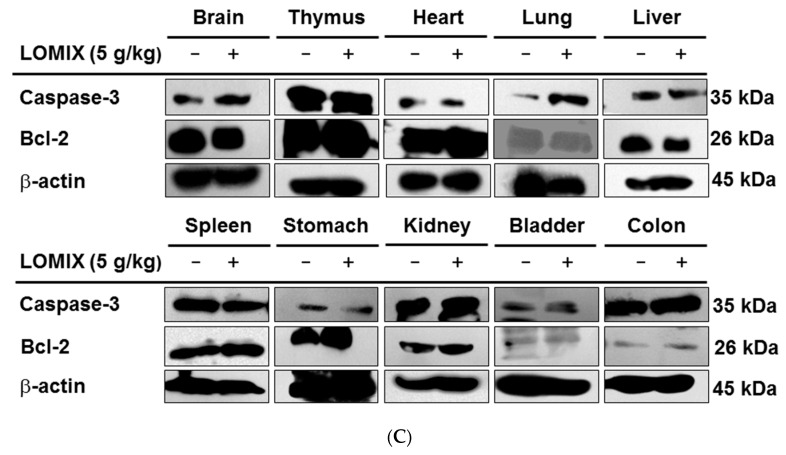
In vivo acute toxicity study of LOMIX. ICR mice received a single dose of LOMIX (5 g/kg) orally, and organs (brain, thymus, heart, lung, liver, spleen, stomach, kidney, bladder, and colon) were excised after 24 h. (**A**) Organs were photographed, and (**B**) the weight of the organs was measured using a scale. (**C**) Caspase-3 and Bcl-2 in whole lysates of each organ were detected by Western blot analysis, and β-actin was used as an internal control.

To assess whether LOMIX was cytotoxic in vivo, mice were injected with a high dose of LOMIX (5 g/kg). Organs were then excised from the mice, and the appearance of these organs was compared with those of vehicle-administered control mice. All organs (brain, thymus, heart, lung, liver, spleen, stomach, kidney, bladder, and colon) from the control and LOMIX-administered mice had a similar appearance, with no signs of alteration or damage (Figure 5A). In addition, no weight change was observed in any of these organs between the control and LOMIX-administered mice (Figure 5B). Thymus and spleen are primary and secondary lymphoid organs, respectively; LOMIX did not appear to be toxic to these organs, suggesting that LOMIX is not acutely toxic to the immune system. The primary sign of liver toxicity and injury is a change in color of the liver tissue to dark red [24]. No difference in color of liver tissues was observed when control and LOMIX-administered mice were compared, indicating that LOMIX does not induce liver injury and toxicity. Colon toxicity results in damage to colon tissues, leading to colon shortening [23,37]. LOMIX did not induce colon shortening, indicating that LOMIX is not acutely toxic to the colon. Together, these results suggest that LOMIX is not acutely toxic in vivo and does not have pathological consequences. In vivo acute toxicity of LOMIX was further investigated by biochemical evaluation. Apoptosis, which refers to programmed cell death, is a molecular control point in toxicity [41] and is characterized by changes in activation and expression of apoptosis-related proteins such as caspases and Bcl-2 family proteins [42]. We evaluated the expression of caspase-3 and Bcl-2, which are proapoptotic and antiapoptotic molecules, respectively, in LOMIX-treated mice to determine if there were biochemical signs of LOMIX toxicity. LOMIX did not induce alterations in protein expression of either caspase-3 or Bcl-2 in any organ (Figure 5C), indicating that LOMIX does not affect the apoptotic process or apoptosis-regulated toxicity. Although LOMIX induced caspase-3 expression in the lung, the reason for this is unclear, and future investigation of this finding is required. These results strongly suggest that LOMIX does not induce in vivo acute toxicity, but further studies, including a dose-escalation study to determine the maximum-tolerated dose, a chronic toxicity study, and a toxicity study in human subjects are needed.

### 3.6. Anti-Inflammatory Effect of Each Linusorb in LOMIX

**Figure 6:**
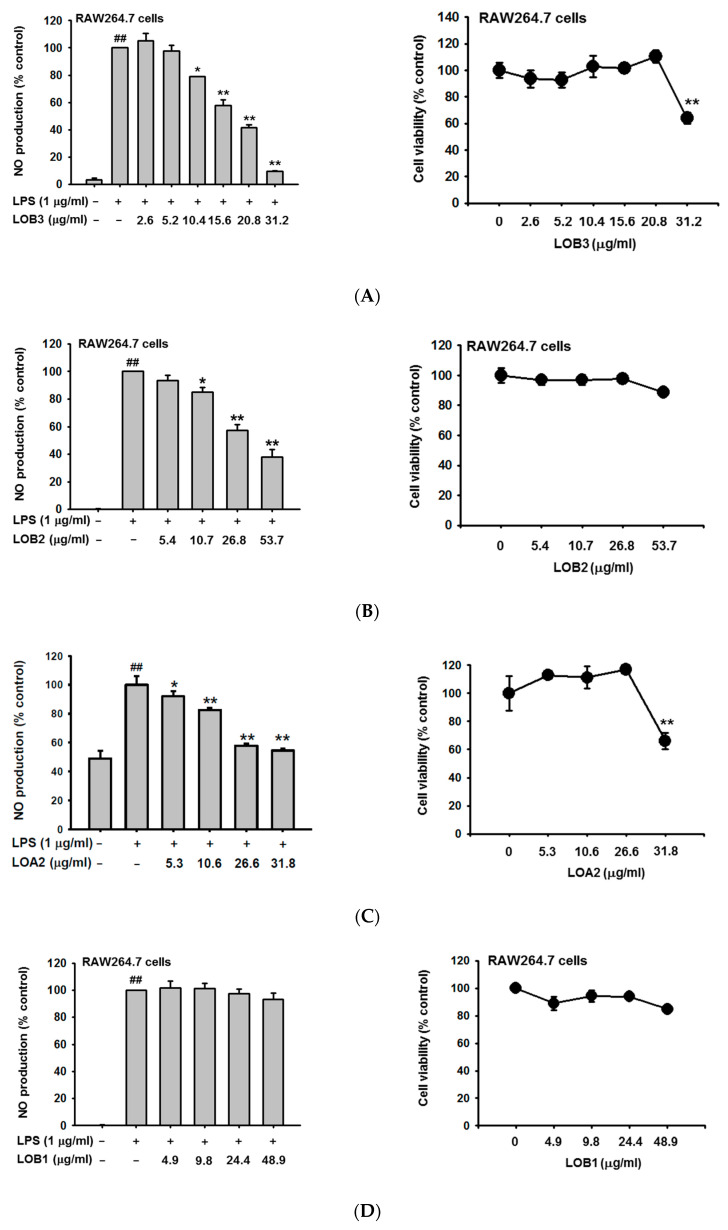

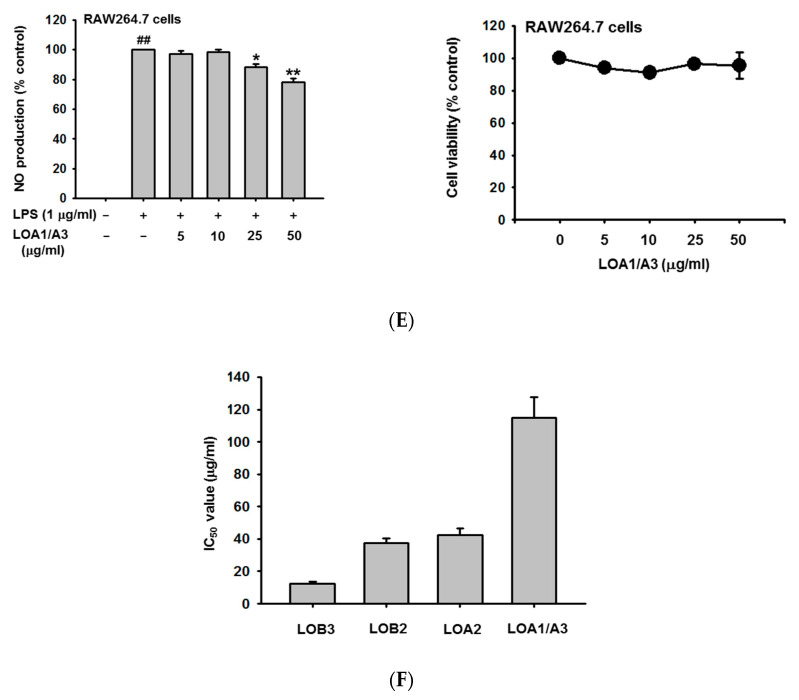
Anti-inflammatory effect of each linusorb in LOMIX. RAW264.7 cells pretreated with the indicated doses of either (**A left**) LOB3, (**B left**) LOB2, (**C left**) LOA2, (**D left**) LOB1, or (**E left**) LOA1/A3 for 30 min were treated with LPS (1 µg/mL) for 24 h, and NO level in the cell culture media was determined by Griess assay. RAW264.7 cells were treated with the indicated doses of either (**A right**) LOB3, (**B right**) LOB2, (**C right**) LOA2, (**D right**) LOB1, or (**E right**) LOA1/A3 for 24 h, and the cell viability was determined by MTT assay. (**F**) IC_50_ values of LOB3, LOB2, LOA2, and LOA1/A3 for NO production. ^##^ *p* < 0.01 compared to the vehicle-treated control. * *p* < 0.05, ** *p* < 0.01 compared to the stimulator-treated controls.

Since LOMIX is a mixture of several linusorbs (Figure 1A and Table 1), the anti-inflammatory effect to inhibit NO production and the cytotoxicity of each linusorb in LOMIX was evaluated in RAW264.7 cells. Doses of each linusorb were decided based on levels of each linusorb found in LOMIX (see Table 2). LOB3 (Figure 6A left), LOB2 (Figure 6B left), and LOA2 (Figure 6C left) significantly inhibited NO production in the LPS-stimulated RAW264.7 cells in a dose-dependent manner, whereas, LOA1/A3 slightly inhibited NO production (Figure 6E left), and LOB1 did not inhibit NO production in the LPS-stimulated RAW264.7 cells (Figure 6D left). Therefore, these results suggest that LOB3, LOB2, and LOA2 are active anti-inflammatory linusorbs included in LOMIX, as assessed by NO assay. IC_50_ values for NO production of each linusorbs were further determined, and those of LOB3, LOB2, LOA2, and LOA1/A3 were 12.5, 37.3, 42.5, and 114.7 µg/mL, respectively (Figure 6F), while LOB1 showed IC_50_ value of >200 µg/mL (data not shown). Interestingly, the amounts of only two linusorbs, LOB3 and LOB2 in LOMIX which showed the suppressive effect on NO production (Figure 1B) were higher than their IC_50_ (Table 1 and Figure 6F), which suggests that the main linusorbs in LOMIX to show anti-inflammatory effect by inhibiting NO production in RAW264.7 cells might be LOB3 and LOB2. Indeed, suppressive effect of LOB3 and LOB2 on NO production was better than that of other linusorbs, LOA2, LOB1, and LOA1/A3 in LPS-stimulated RAW264.7 cells (Figure 6A–E right).

The cytotoxicity of each linusorb in LOMIX was next evaluated in RAW264.7 cells. No cytotoxicity was observed in LOB2 (Figure 6B right), LOA2 (Figure 6C right), LOB1 (Figure 6D right), and LOA1/A3 (Figure 6E right), whereas, LOB3 showed the cytotoxicity at a high dose (31.2 µg/mL) in RAW264.7 cells (Figure 6A right). Interestingly, although LOB3 showed cytotoxicity at 31.2 µg/mL (Figure 6A right), LOMIX containing a higher dose of LOB3 (45.9 µg/mL, Table 1) than 31.2 µg/mL did not show cytotoxicity in RAW264.7 cells (Figure 1C). The reason for these results is unclear, however, this observation might be explained that despite higher dose of LOB3 in LOMIX (45.9 µg/mL) than 31.2 µg/mL, LOB3 in total LOMIX is 22.95% (Table 2), which might compensate the cytotoxicity induced by LOB3 alone.

## 4. Conclusions

**Figure 7:**
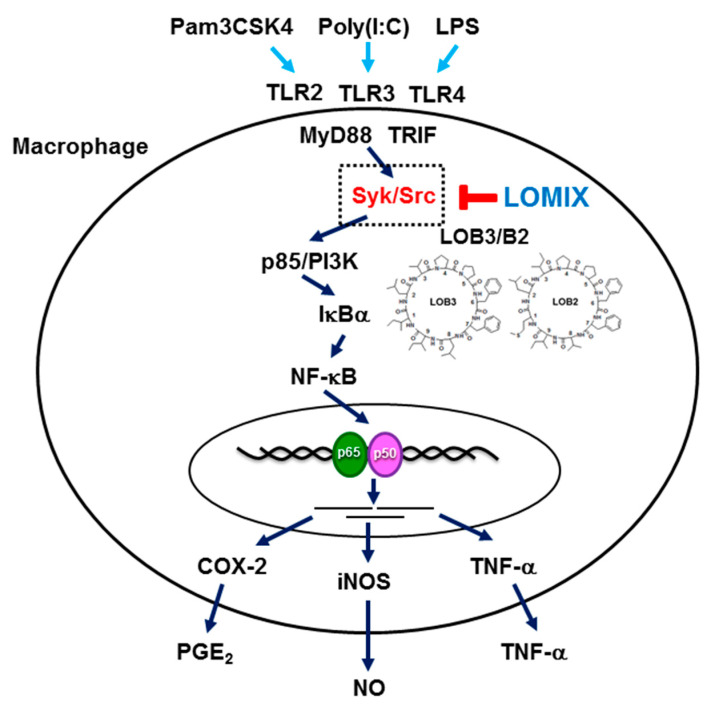
Schematic summary describing LOMIX-mediated anti-inflammatory activity in macrophages.

We investigated the anti-inflammatory effects of LOMIX both in vitro and in vivo. The results clearly showed that LOMIX had an in vitro anti-inflammatory effect by inhibiting the production of inflammatory mediators and expression of inflammatory genes by targeting Src and Syk in the NF-κB signaling pathway. LOMIX also had an in vivo anti-inflammatory effect in several inflammatory disease animal models by ameliorating the symptoms of these diseases. Although LOMIX exerted a strong anti-inflammatory effect, LOMIX did not exhibit toxicity, either in vitro or in vivo. Moreover, LOB3, LOB2, and LOA2 in LOMIX showed the strong anti-inflammatory effect by inhibiting NO production. Taken together, these results suggest that LOMIX exerts an anti-inflammatory effect by targeting the NF-κB signaling pathway (Figure 7) and has the potential to be developed as a novel anti-inflammatory medication to prevent and treat various inflammatory diseases.

## Figures and Tables

**Table 1:** Primer sequences used for polymerase chain reaction (PCR).

Target		Sequence (5′ to 3′)
**Quantitative Real-Time PCR**		
iNOS	Forward	GGAGCCTTTAGACCTCAACAGA
	Reverse	TGAACGAGGAGGGTGGTG
COX-2	Forward	GGGAGTCTGGAACATTGTGAA
	Reverse	GCACATTGTAAGTAGGTGGACTGT
TNF-α	Forward	TGCCTATGTCTCAGCCTCTTC
	Reverse	GAGGCCATTTGGGAACTTCT
GAPDH	Forward	CAATGAATACGGCTACAGCAAC
	Reverse	AGGGAGATGCTCAGTGTTGG
**Semiquantitative RT-PCR**		
iNOS	Forward	CCCTTCCGAAGTTTCTGGCAGCAG
	Reverse	GGCTGTCAGAGCCTCGTGGCTTTGG
COX-2	Forward	CACTACATCCTGACCCACTT
	Reverse	ATGCTCCTGCTTGAGTATGT
TNF-α	Forward	TTGACCTCAGCGCTGAGTTG
	Reverse	CCTGTAGCCCACGTCGTAGC
GAPDH	Forward	CACTCACGGCAAATTCAACGGCAC
	Reverse	GACTCCACGACATACTCAGCAC

**Table 2:** The composition of linusorb mixture (LOMIX) by HPLC.

Code	Linusorb ^a^	MW	Quantity ^b^	Amount (µg/mL) ^c^
LOB3	[1-9-NαC]-linusorb B3	1040.34	0.14 mg (22.95%)	45.9
LOB2	[1-9-NαC]-linusorb B2	1074.37	0.16 mg (26.23%)	52.5
LOA2	[1-8-NαC], [1-(*R*_s_,*S*_s_)-MetO]-linusorb A2	1064.34	0.05 mg (8.20%)	16.4
LOB1	[1-8-NαC], [1-(*R*_s_,*S*_s_)-MetO]-linusorb B1	977.26	0.12 mg (19.67%)	39.3
LOA3	[1-8-NαC], [1,3-(*R*_s_,*S*_s_)-MetO]-linusorb A3	1084.35	0.03 mg (4.92%)	45.9
LOA1	[1-8-NαC], [1,3-(*R*_s_,*S*_s_)-MetO]-linusorb A1	1098.38	0.11 mg (18.03%)	

^a^ All linusorbs listed are analyzed by Shim et al. [43], ^b^ percent and quantity of each linusorb in total LOMIX, and ^c^ amount of each linusorb in LOMIX (200 µg/mL) relative to the stated quantity (%).
